# Criticality and universality in neuronal cultures during “up” and “down” states

**DOI:** 10.3389/fncir.2024.1456558

**Published:** 2024-09-10

**Authors:** Mohammad Yaghoubi, Javier G. Orlandi, Michael A. Colicos, Jörn Davidsen

**Affiliations:** ^1^Complexity Science Group, Department of Physics and Astronomy, Faculty of Science, University of Calgary, Calgary, AB, Canada; ^2^Integrated Program in Neuroscience, McGill University, Montreal, QC, Canada; ^3^Hotchkiss Brain Institute, University of Calgary, Calgary, AB, Canada; ^4^Department of Physiology and Pharmacology, Faculty of Medicine, University of Calgary, Calgary, AB, Canada

**Keywords:** up and down states, critical brain dynamics, neuronal avalanches, dissociated cultures, self-organization mechanisms in neuronal systems

## Abstract

The brain can be seen as a self-organized dynamical system that optimizes information processing and storage capabilities. This is supported by studies across scales, from small neuronal assemblies to the whole brain, where neuronal activity exhibits features typically associated with phase transitions in statistical physics. Such a critical state is characterized by the emergence of scale-free statistics as captured, for example, by the sizes and durations of activity avalanches corresponding to a cascading process of information flow. Another phenomenon observed during sleep, under anesthesia, and in *in vitro* cultures, is that cortical and hippocampal neuronal networks alternate between “up” and “down” states characterized by very distinct firing rates. Previous theoretical work has been able to relate these two concepts and proposed that only up states are critical whereas down states are subcritical, also indicating that the brain spontaneously transitions between the two. Using high-speed high-resolution calcium imaging recordings of neuronal cultures, we test this hypothesis here by analyzing the neuronal avalanche statistics in populations of thousands of neurons during “up” and “down” states separately. We find that both “up” and “down” states can exhibit scale-free behavior when taking into account their intrinsic time scales. In particular, the statistical signature of “down” states is indistinguishable from those observed previously in cultures without “up” states. We show that such behavior can not be explained by network models of non-conservative leaky integrate-and-fire neurons with short-term synaptic depression, even when realistic noise levels, spatial network embeddings, and heterogeneous populations are taken into account, which instead exhibits behavior consistent with previous theoretical models. Similar differences were also observed when taking into consideration finite-size scaling effects, suggesting that the intrinsic dynamics and self-organization mechanisms of these cultures might be more complex than previously thought. In particular, our findings point to the existence of different mechanisms of neuronal communication, with different time scales, acting during either high-activity or low-activity states, potentially requiring different plasticity mechanisms.

## 1 Introduction

The description of neuronal dynamics within the framework of critical phenomena has become commonplace amongst physicists in the last decade or so (Chialvo, 2010; Massobrio et al., 2015; O'Byrne and Jerbi, 2022). The first signatures of criticality in the brain were depicted as neuronal avalanches in organotypic cortical cultures (Beggs and Plenz, 2003, 2004), where sequences of high-frequency neuronal activations across a small population could be described by scale-free statistics of their sizes and durations distributions. Nowadays, similar critical signatures have been observed across many systems and preparations: from power-law statistics of correlations in whole-brain recordings (Tagliazucchi et al., 2012; Haimovici et al., 2013; Ponce-Alvarez et al., 2018) to neuronal avalanches in slices (Gireesh and Plenz, 2008), dissociated cultures (Pasquale et al., 2008; Yaghoubi et al., 2018), and *in vivo* (Petermann et al., 2009; Bellay et al., 2015; Yu et al., 2017; Priesemann et al., 2013; Curic et al., 2021; Rabus et al., 2023). They indicate the emergence of complex spatiotemporal dynamics with statistics compatible with a system being in the neighborhood of a critical point, in particular, near a second-order phase transition, or of a critical region (Moretti and Muñoz, 2013).

From a statistical physics point of view, neuronal avalanches are interpreted as a branching process (Beggs and Plenz, 2003; Korchinski et al., 2021), where an active neuron has a finite probability of activating its neighbors. When each active neuron induces, on average, the firing of a single neighbor, the system is thought to be in a critical state, where the activity neither explodes nor always dies out quickly. This results in a scale-free distribution of activation sequences observables, namely the size and duration of the neuronal avalanches. A key assumption in this picture is that there exists a separation of time scales, where the spontaneous activations of neurons (those that initiate an avalanche) happen much more slowly than the spreading of that avalanche across the population, i.e., there only exists a single avalanche at any given time. However, in most neuronal systems, that is far from the truth (Orlandi et al., 2013; Williams-Garćıa et al., 2017), and many avalanches can coexist simultaneously, resulting in a more difficult interpretation of the observed statistics (Korchinski et al., 2021).

Until recently (Yaghoubi et al., 2018; Yu et al., 2017), neuronal avalanches had mostly been described in systems where the network switches between periods of high and low-frequency activity such that the periods of high activity dominated the neuronal avalanche statistics. *In vivo* and in some slice preparations, such a switching behavior corresponds to the so-called “up” and “down” states, i.e., slow cortical oscillations present during slow-wave sleep (Sanchez-Vives and McCormick, 2000) that are initiated by pyramidal neurons near layer V and propagate toward the other cortical layers. In other preparations, like dissociated cultures (Orlandi et al., 2013), the bistable behavior is more akin to hippocampal sharp wave-ripples (Levenstein et al., 2018), which also have a well-defined size and duration. Theoretically, this phenomenon has often been described in terms of synchronization (Penn et al., 2016) or as spontaneous switching around a bifurcation (Millman et al., 2010). However, both cortical up and down states and hippocampal short-wave ripples have a strong spatial component, initiating at specific sites and propagating throughout the tissue, reminiscent of a classical spatially-extended excitable system (Orlandi and Casademunt, 2017).

The specific hypothesis of spontaneous switching around a bifurcation implies that the dynamics in the high-activity state are critical whereas it is subcritical in the low-activity state (Millman et al., 2010). Here, we test this hypothesis explicitly in neuronal cultures that do show alternating activity behavior by analyzing the two different states separately. We find that both high-activity and low-activity states can exhibit similar critical signatures if appropriate time scales are chosen for defining neuronal avalanches. These results point to the existence of different mechanisms of neuronal communication, with different time scales, acting during either high-activity or low-activity states. We also show that detailed model simulations of dissociated cultures follow the aforementioned hypothesis and are, thus, incompatible with the experimental observations for the low-activity state. This suggests the existence of processes with long timescales (of the order of a few hundred ms) that play a significant role in shaping the dynamics during the low-activity state that are currently not captured by existing models.

## 2 Materials and methods

### 2.1 Hippocampal cultures

Cultures from dissociated hippocampal neurons and glial cells, prepared from newborn P0 Sprague-Dawley rats, were plated on Si chips of 1 mm thickness, and 1 cm^2^ surface area, placed on individual 24-well plate wells; as described previously (Colicos et al., 2001; Girotto et al., 2013). Each chip was Matrigel-coated (Beckton Dickinson) and placed in Basal Medium Eagle (BME). Cells were initially plated at a density of 30,000 cells/ml. The culture medium was not changed during the first week and every 4 days thereafter.

### 2.2 Calcium imaging

Cultures were grown for up to 2–3 weeks before imaging. Prior to imaging, cultures were incubated with Fluo-4 calcium indicator for 20 min. Afterward, cultures were washed and placed in an individual well with an extracellular bath solution (EBS) containing 135 mM NaCl, 10 mM glucose, 3 mM CaCl_2_, 5 mM KCl, 2 mM MgCl_2_ and 5 mM Hepes, pH was adjusted to 7.3 with NaOH, and osmolarity to 310 mOsm with Sorbital. Calcium imaging was performed following References (Colicos et al., 2001; Yaghoubi et al., 2018). In brief, we used a high temporal resolution camera that allowed us to record neuronal activity at 200 fps (Hammamatsu Orca-Flash 4.0) on an upright microscope with low magnification (field of view of *L*≈500 μm). We recorded the spontaneous, non-stimulated fluorescence neuronal activity of up to 1,500 neurons for 20 min (pixel resolution of 0.65μm/pixel). With this setup we are able to record most neuronal activity within a large field of view with single-cell resolution and a temporal resolution comparable to the fastest time-scales of synaptic integration; avoiding several of the drawbacks caused by spatial and temporal sub-sampling and hidden neurons (Ribeiro et al., 2014; Levina and Priesemann, 2017).

### 2.3 Pharmacology

In a subset of experiments, we blocked inhibitory connections by performing bath application of picrotoxin (PTX), a noncompetitive GABA_A_ receptor antagonist, with a concentration of 50 μM during 15 min at room temperature after the first imaging session. Cultures were then typically imaged in a different field of view for an additional 20 min.

### 2.4 Data preprocessing

**Figure 1:**
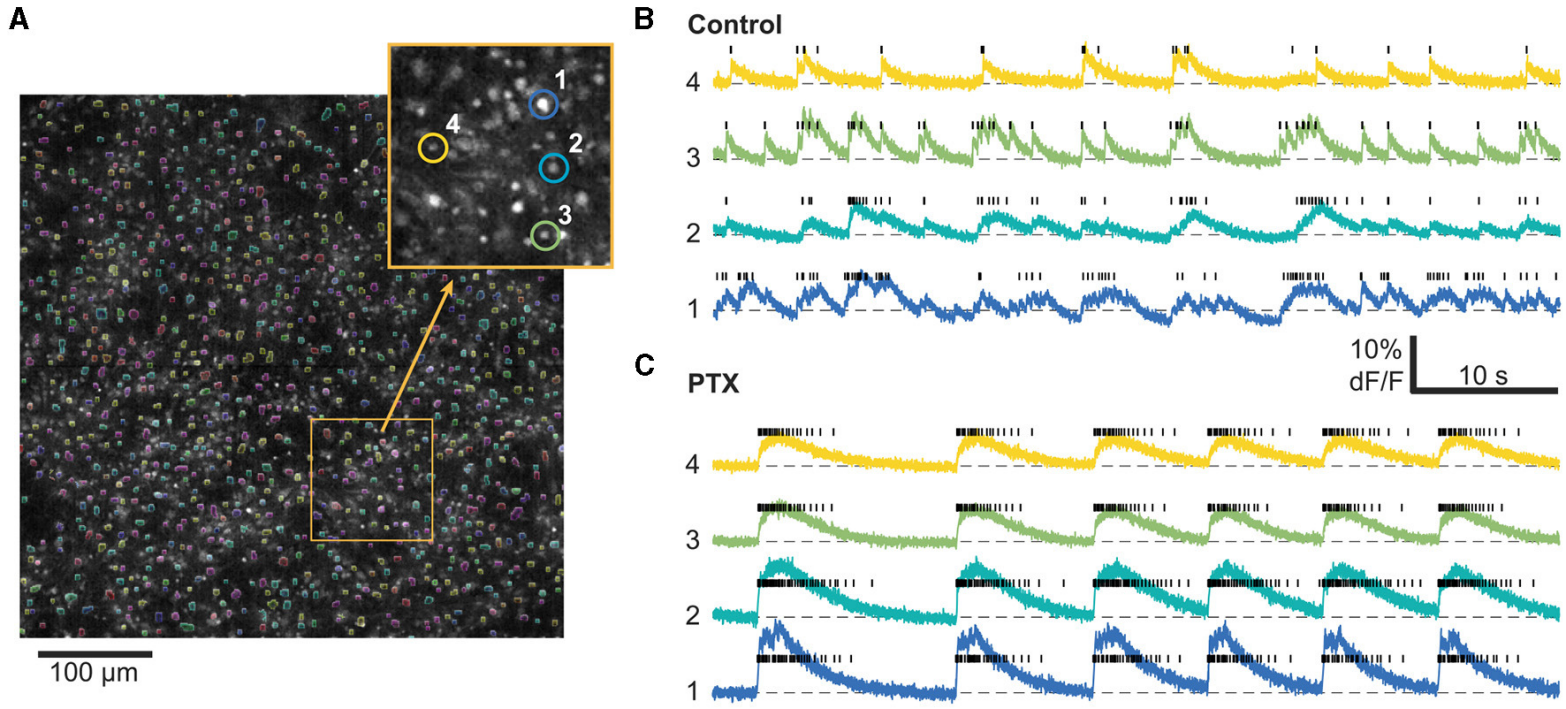
Calcium imaging analysis workflow. **(A)** 1,000–1,500 cells are automatically segmented within the field of view based on their average fluorescence. Inset: Zoom in on the field of view with four characteristic cells highlighted. Right: Timeseries from the same 4 highlighted cells as well as their inferred spike trains (black bars) before **(B)** and after **(C)** PTX application. For this particular example, we imaged the same cells before and after PTX. Only a smaller time window out of the 20 min recordings is shown.

Data preprocessing of calcium imaging experiments was performed as previously described in Fernández-García et al. (2020) using the NETCAL software (Orlandi et al., 2017) platform (see Figure 1). In brief, cell ROIs were automatically detected using a simple thresholding procedure on the time-averaged image of the recording and posteriorly cleaned up with morphological opening operations. Time series for each ROI were extracted, detrended, and normalized to Δ*F*/*F*_0_ units (where *F*_0_ was computed prior to the detrending operation). Spike inference was performed using the OASIS algorithm (Friedrich et al., 2017). See Table 1 for a summary of the list of recording experiments and their properties.

**Table 1:** Summary of the properties of different recordings.

**Recording #**	**Type**	**# Cells**	**〈ISI〉_up_ (ms)**	**〈ISI〉_down_ (ms)**
1	E + I	1,374	216	2,955
2	E + I	1,510	211	1,438
3	E	1,426	210	419
4	E + I	825	86	449
5	E	813	407	1,444
6	E + I	998	70	245
7	E	1,001	248	613
8	E + I	893	106	620
9	E	875	404	1409

This includes the presence (E + I) or absence (E) of active inhibitory cells as well as the average interspike interval (ISI) per neuron 〈ISI〉 during the up and down states.

### 2.5 Mathematical model and simulations

The dynamics of cultures from dissociated neurons were modeled and simulated following (Orlandi et al., 2013; Orlandi and Casademunt, 2017). In brief, for the 'homogeneous' simulations (simulations 1 and 2), single neuron dynamics are modeled by a quadratic integrate and fire model with adaptation (Alvarez-Lacalle and Moses, 2009; Izhikevich, 2003, 2007), i.e.,


(1) $$\begin{array}{ll} C\dot{v} & =k\left(v-v_{r}\right)\left(v-v_{t}\right)-u+I+I_{0}+\eta , \end{array}$$

(2) $$\begin{array}{ll} \tau_{a}\dot{u} & =b\left(v-v_{r}\right)-u, \end{array}$$

(3) $$\begin{array}{ll} \text{if }v\geq v_{p}, & \text{then }v\leftarrow v_{c},u\leftarrow u+d. \end{array}$$

Here, Equation 1 corresponds to the dynamics of the soma membrane potential *v*(*t*) and *v*_*r*_ = −60 mV and *v*_*t*_ = −40 mV are the resting and threshold potentials, respectively. *C* = 100 ms is the normalized membrane capacitance and *u* models an inhibitory current that represents internal slow currents generated by the activation of ion channels. *I* accounts for the synaptic inputs from other neurons. *I*_0_ represents the spontaneous release of synaptic vesicles (minis). The time between successive minis is often modeled as a memoryless, exponential process (Fatt and Katz, 1952). Hence, the number of minis released in a given interval will follow a Poisson process. The rate of this process depends on the experimental conditions (Ivenshitz and Segal, 2010), but in our simulations, and following previous work (Orlandi et al., 2013), we selected a frequency of λ = 0.1 ms^−1^, which is chosen to produce a spontaneous firing rate of the neurons of the order of 0.1 Hz. Each mini generates a current with amplitude *g*_*m*_ = 30 mV which decays exponentially with a time constant τ_*m*_ = 10 ms. η is a white noise term with autocorrelation $\langle \eta \left(t\right)\eta \left(t^{\prime }\right)\rangle =2g_{s}\delta \left(t-t^{\prime }\right)$ with *g*_*s*_ = 30 mV. Equation 2 is the evolution of the slow currents and τ_*a*_ = 33 ms, *k* = 0.7 mV^−1^, *b* = −2 and *d* = 100 mV are parameters that control recovery and adaptation.

Every time a given neuron *i* fires, it produces an (excitatory or inhibitory) current on its output neighbors of the form


(4) $$\begin{array}{l} I_{i}\left(t,t_{m}\right)=gD_{i}\left(t_{m}\right)\exp\left(\frac{t-t_{m}}{\tau }\right)\text{Θ}\left(t-t_{m}\right) \end{array}$$

where *t*_*m*_ is the spike time and *g* is the synaptic strength. Its value for excitatory synapses is used as a control parameter to obtain the desired time interval separating subsequent up states (which is achieved for *g*≈40 mV) while the inhibitory strength is fixed at −50 mV. τ is the characteristic time constant (10 ms for excitatory currents and 20 ms for inhibitory ones). Θ(*t*) is the Heaviside function and *D* short-term synaptic depression (STD). The evolution of STD is described by


(5) $$\begin{array}{l} Ḋ=\frac{1}{\tau_{D}}\left(1-D\right)-\left(1-\beta \right)D\delta \left(t-t_{m}\right), \end{array}$$

where τ_*D*_ (2 s for excitatory and 0.2 s for inhibitory cells), is the synaptic recovery time and β (0.8 for excitatory and 0.95 for inhibitory cells) controls the level of depression after each spike.

To mimic the experimental cultures and establish realistic connectivity patterns, neurons were placed randomly on a square region with 10 mm sidelength until a density of ρ = 800 neurons/mm^2^ was reached (80,000 neurons total). For each neuron, an axon was grown as a biased random walk with total length given by a Rayleigh distribution with variance 900 μm^2^. Starting from the cell body a starting angular direction was picked randomly and a segment of 10 μm was grown. At the end of the segment, a new direction was chosen centered on the previous one following a Gaussian distribution with a standard deviation of 15 °. This process was repeated until the desired total length was reached. For each neuron, a dendritic tree was modeled as an effective circular area of interaction with a radius obtained from a Gaussian distribution with mean 150 μm and standard deviation 40 μm. If an axon crossed the dendritic tree of another neuron, a connection was established with probability 0.13. Finally, 20 % of the neurons were randomly chosen to be inhibitory and the remaining ones excitatory.

For the “heterogeneous” simulations (simulations 3 and 4) we lowered the standard excitatory population to 70% and added 10% of excitatory bursty cells (accomplished by changing *v*_*c*_ = −40 and *d* = 50). To all excitatory cells, we changed the resting membrane potential to a base value of *v*_*r*_ = −62 mV and added an offset drawn from a Rayleigh distribution with standard deviation σ = 2 mV, to add variability to the firing rates consistent with experimental data.

Each simulation had a fixed run length of 1 h and was simulated with a first-order Euler algorithm with a time step of 0.1 ms. Random numbers were generated with the MTGP32 implementation of the Mersenne Twister for the GPU (Saito and Matsumoto, 2013) and initialized with a random seed for each simulation. In total, we ran simulations in 4 different conditions: simulations 1 and 2 corresponded to homogeneous networks, and 3 and 4 to heterogeneous ones. In simulations 2 and 4 we mimicked the effect of blocking inhibition by setting the strength of inhibitory connections to 0.

### 2.6 Detection of up and down states

The detection of up and down states is done based on thresholding the population firing rate. The firing rate for each frame is defined as the number of spikes in that frame normalized by the number of neurons. A Gaussian kernel with σ= 5 frames for experimentally recorded data and σ= 20 frames for simulated data is used to smooth the firing rate traces. To separate up and down states we used a Schmitt trigger (Taub and Schilling, 1977), thresholding the normalized activity with an upper threshold = 0.001 and a lower threshold = 0.0003. We kept the same criteria for all recordings and simulations.

### 2.7 Neuronal avalanches and scaling collapse procedure

Following the standard approach for spiking data (Friedman et al., 2012; Pasquale et al., 2008), a neuronal avalanche is defined as the largest sequence of consecutive time bins containing spikes in every single time bin, separated by time bins during which none of the neurons in the culture fire (see Supplementary Figure S1 for some examples). The avalanche duration, *T*, corresponds to the number of time bins and the avalanche size, *S*, is the total number of spikes over the duration of an avalanche. Based on this definition, for a fixed number of neurons, it is expected that the choice of the size of the time bin affects the avalanche statistics, specifically the size *S* and duration *T* of avalanches. To capture this, we follow here the standard finite size scaling approach used in the context of phase transitions (see, for example, Christensen and Moloney, 2005). In our context, its original formulation in terms of a varying number of neurons can be recast in terms of a varying size of the time bin. It is based on the hypothesis that for a fixed number of neurons, the effect of temporal bin size on scale-free avalanche statistics (e.g., avalanche size distribution, *P*(*S*)) can be taken into account as follows:


(6) $$\begin{array}{l} \text{P}\left(\text{S}\right)\sim {\text{S}}^{-\tau }\times \text{f}\left(\text{S}/{\text{bin}}^{\beta_{S}}\right). \end{array}$$

This functional form indicates, for suitably chosen scaling exponents τ and β_*S*_, the distributions for different bin sizes can be collapsed onto the scaling function *f* by plotting P(S) × S^τ^ vs $\text{S}/{\text{bin}}^{\beta_{S}}$. In the case of exponential-like avalanche statistics (τ = 0), one can achieve a similar data collapse with a scaling exponent β_*S*_ when plotting $\text{P}\left(\text{S}\right)\times {\text{bin}}^{\beta_{S}}$ vs $\text{S}/{\text{bin}}^{\beta_{S}}$. In both cases, to numerically determine the values of the scaling exponent(s) the distinctive features of each graph need to align horizontally for the largest arguments aka the right-hand side of the graph (Christensen and Moloney, 2005), which is typically assessed by eye as we do here and allows us to estimate the uncertainties.

To replicate the experimental field of view (which covers only a small fraction of the whole culture) in the simulations, we only analyzed the avalanche statistics of neurons within a circular patch of radius *r* = 0.5 mm for the *E* simulations and *r* = 0.8 mm in the E + I simulations. This radius was chosen to ensure that the total activity rate in the up states was of the order of 1 event per frame or time step (0.1 ms). Each of these patches contained in total 400 to 1,000 neurons. To increase the number of avalanches and obtain reliable estimates of the avalanche statistics, for each simulation and each experiment, we randomly selected 100 different local patches consisting of 50% of the neurons and combined the avalanches from all patches into a single distribution. This approach also allows us to obtain avalanche statistics for different temporal bin sizes.

### 2.8 *p*-value estimation for power-law distributions

After identifying the optimal exponents α and β, using the scaling collapse procedure (Equation 6), our next step is to assess the validity of the power-law model as a hypothesis for the specific recording or simulation. To achieve this, we check whether we can identify an extended range for the size or duration in the avalanche distribution function that gives a reasonably high *p*-value (>0.1). A detailed description of the process of identifying the range is presented in the captions of Supplementary Figure S2. To find the *p*-value, we first used the Kolmogorov-Smirnov (KS) statistic. KS statistic quantifies the distance between two probability distributions (Press et al., 2007), which is defined as the maximum distance between the cumulative distribution functions (CDFs) of the two distributions (here data and fitted model):


(7) $$\begin{array}{l} d_{e}=\max|S_{e}\left(x\right)-P_{e}\left(x\right)|,\;X_{low}\leq x\leq X_{high} \end{array}$$

Here, *S*_*e*_(*x*) is the CDF of the empirical data, and *P*_*e*_(*x*) is the CDF corresponding to the fitted model. The fitted model is estimated using the power-law scaling procedure related to Equation 6. The reported *p*-value is the probability of observing a KS value bigger than *d*_*e*_ for synthetic data generated by the fitted model and provides a measure of whether it is likely that the empirical data do indeed follow the fitted model. One can show that its value can be calculated from the following theoretical expression (Deluca and Corral, 2013):


(8) $$\begin{array}{l} \text{p}-\text{value}=2\overset{\infty }{\underset{i=1}{\sum }}{\left(-1\right)}^{i-1}\exp[-2i^{2}{\left(d_{e}\sqrt{n}+0.12d_{e}+0.11d_{e}/\sqrt{n}\right)}^{2}], \end{array}$$

where *n* is the number of samples in the data set. As mentioned above, to enhance our statistics we identified avalanches over 100 iterations (for both experimental and simulation data). The reported *p*-value is calculated over each of those iterations. The reported *p*-value is the mean ± standard error of the mean (SEM), where the mean value and SEM are calculated over 100 iterations. See Supplementary Figure S2 for a representative example of the procedure and Supplementary Table S2 for fitting details.

## 3 Results

### 3.1 Neuronal cultures

**Figure 2:**
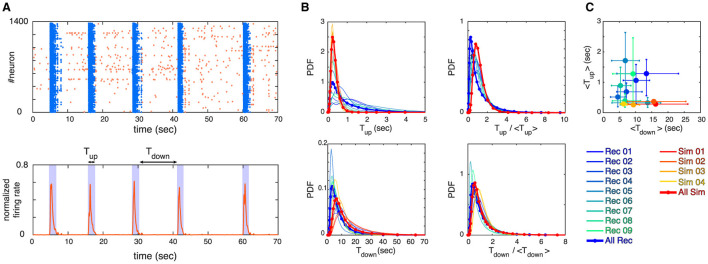
**(A)** Example of a raster plot for an experimental recording (top panel) and its corresponding normalized firing rate (bottom panel) are depicted. The up and down states are identified by thresholding the firing rate, where bins of data with firing rate > threshold are identified as up state and bins of data with firing rate less threshold are identified as down state (see section 2 for details). We define *T*_up_ and *T*_down_ to measure the lengths of up and down states as visualized in the bottom panel. **(B)** Probability Density Functions (PDFs) of *T*_up_, *T*_down_, *T*_up_/ < *T*_up_>, and *T*_down_/ < *T*_down_> are shown, where each curve represents one of the experimental recordings or simulations (see legends). **(C)** The mean values of *T*_up_ and *T*_down_ for all the recordings and simulations are shown. Error bars correspond to 75 percentiles and the same color coding as panel **(B)** is used.

The overall activity of two to three-week-old neuronal cultures typically switches between high activity periods or “up states” and low activity periods or ‘down states‘ as shown in Figure 2. The up states—often also referred to as network bursts—occur quasi-periodically and involve the vast majority of all neurons. As Figure 2 also shows, their duration is quite regular as well. The activity during up states can be understood as a set of causal cascades of induced firings across the observed population of neurons in the culture, often modeled as a branching process (Beggs and Plenz, 2003). The cascade of neuronal activity is studied in the framework of neuronal avalanches as described in the Section 2. Previous studies have found that neuronal avalanches exhibit statistics of a branching process at or close to its critical point (Beggs and Plenz, 2003; Friedman et al., 2012). In the often-observed case of a mean-field branching process, the activity propagates on a tree-like network without feed-back loops and the avalanches follow a scale-free behavior, i.e.,


(9) $$\begin{array}{l} P\left(S\right)\sim S^{-\tau } \end{array}$$

(10) $$\begin{array}{l} P\left(T\right)\sim T^{-\alpha } \end{array}$$

(11) $$\begin{array}{l} \langle T\rangle \left(S\right)\sim S^{\gamma } \end{array}$$

where *P* is the probability distribution function (PDF) of the associated variable and τ = 1.5, α = 2.0, and γ = 0.5 are the critical mean-field exponents (Friedman et al., 2012). These critical exponents—whether they take on mean-field values or not—are necessarily related through the scaling relation


(12) $$\begin{array}{l} \left(\alpha -1\right)/\left(\tau -1\right)=\gamma^{-1}, \end{array}$$

which provides an additional test for the presence of critical behavior.

**Figure 3:**
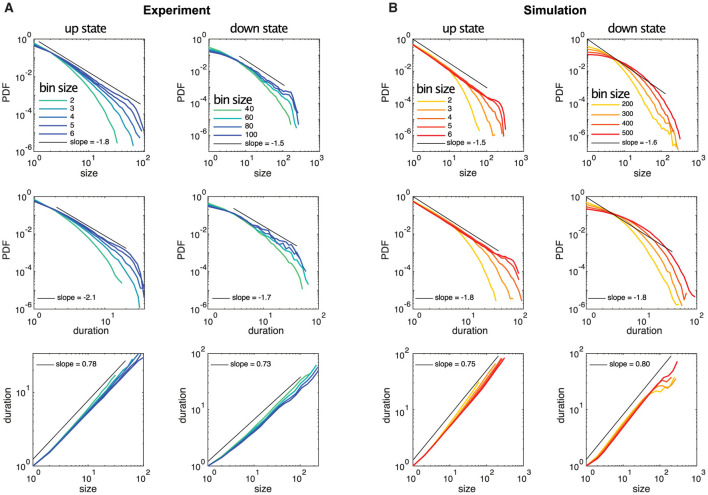
Example distributions of neuronal avalanche sizes and durations for up and down states of experimental data and simulated data are plotted for different temporal bin sizes, where the bin size of 1 corresponds to the temporal resolution of the recordings (5 ms) and the simulations (0.1 ms), respectively. **(A)** The up state is from experimental recording 3, the down state is from recording 1. **(B)** Simulation 1 is shown but all simulations exhibit almost identical avalanche statistics.

Here, we study the avalanche statistics of the up state and the down state separately. Due to the vastly different activity levels, the corresponding ISIs per neuron are also vastly different. For our experimentally recorded data the single cell ISIs are: ISI_up state_ = 217ms ± 43 ms and ISI_down state_ = 1, 065ms ± 302ms (mean ± SEM), as follows from Table 1. Nevertheless, we find similar statistical features across up and down states if the different activity levels are taken into account by choosing the time bins to define neuronal avalanches appropriately, being significantly larger for the down state. Examples of distributions of avalanche sizes and durations as well as the relation between sizes and durations are plotted in Figure 3 (see also Supplementary Figures S3–S5). Indeed, for both the up and down state in Figure 3, we recover Equations 9, 10, 11 and the exponents are close to the mean-field values. As Table 2 shows (see also Figure 3), this is often even true for the up state when we block all inhibitory connections by the application of a saturating concentration (50 μM) of picrotoxin (see Section 2 for details). Overall, we find that the majority of experimental recordings have an up state that is consistent with power-law statistics in their sizes over an extended range (see Supplementary Table S1). Such power-law behavior is slightly less prevalent in the down state (Table 2, Supplementary Table S1), but still prominent. Moreover, in all cases the scaling relation (Equation 12) holds within the statistical uncertainties (Supplementary Figure S6), consistent with critical behavior.

**Table 2:** Critical exponents for all of the recordings and simulations.

	**Fitted curve**	**τ**	**β_*S*_**	**α**	**β_*T*_**	**γ**
**Experiments: up state**						
Recording 1	Power-law	1.3 ± 0.1	1.0 ± 0.2	1.8 ± 0.2	0.3 ± 0.1	0.48 ± 0.09
Recording 2	Power-law	1.6 ± 0.1	0.6 ± 0.1	2.1 ± 0.2	0.4 ± 0.1	0.41 ± 0.06
Recording 3	Power-law	1.8 ± 0.1	1.5 ± 0.1	2.1 ± 0.1	1.2 ± 0.1	0.78 ± 0.01
Recording 4	Power-law	1.5 ± 0.1	0.3 ± 0.1	1.9 ± 0.2	0.3 ± 0.1	0.54 ± 0.04
Recording 5	None	NA	NA	NA	NA	0.76 ± 0.04
Recording 6	None	NA	NA	NA	NA	0.56 ± 0.01
Recording 7	None	NA	NA	NA	NA	0.75 ± 0.04
Recording 8	None	NA	NA	NA	NA	0.63 ± 0.02
Recording 9	Power-law	1.7 ± 0.1	1.1 ± 0.1	1.9 ± 0.1	0.5 ± 0.1	0.77 ± 0.05
**Experiments: down state**						
Recording 1	Power-law	1.5 ± 0.1	1.8 ± 0.1	1.7 ± 0.1	1.2 ± 0.1	0.73 ± 0.02
Recording 2	Power-law	1.3 ± 0.1	1.5 ± 0.2	1.6 ± 0.1	0.8 ± 0.2	0.72 ± 0.02
Recording 3	Exponential	NA	0.7 ± 0.1	NA	0.2 ± 0.1	0.51 ± 0.07
Recording 4	Exponential	NA	0.8 ± 0.1	NA	0.5 ± 0.1	0.78 ± 0.05
Recording 5	Power-law	1.1 ± 0.1	1.0 ± 0.1	1.2 ± 0.1	0.6 ± 0.1	0.70 ± 0.09
Recording 6	Exponential	NA	1.1 ± 0.2	NA	0.6 ± 0.1	0.80 ± 0.05
Recording 7	Exponential	NA	1.1 ± 0.1	NA	0.7 ± 0.1	0.71 ± 0.05
Recording 8	Exponential	NA	0.8 ± 0.1	NA	0.5 ± 0.1	0.80 ± 0.06
Recording 9	Exponential	NA	1.3 ± 0.1	NA	1.0 ± 0.1	0.75 ± 0.05
**Simulations: up state**						
Simulation 1	Power-law	1.5 ± 0.1	2.4 ± 0.1	1.8 ± 0.1	2.0 ± 0.1	0.75 ± 0.01
Simulation 2	Power-law	1.6 ± 0.1	2.6 ± 0.1	1.8 ± 0.1	2.0 ± 0.1	0.74 ± 0.01
Simulation 3	Power-law	1.6 ± 0.1	2.4 ± 0.1	1.9 ± 0.1	2.2 ± 0.1	0.75 ± 0.01
Simulation 4	Power-law	1.6 ± 0.1	2.4 ± 0.1	1.8 ± 0.1	1.9 ± 0.1	0.73 ± 0.02
**Simulations: down state**						
Simulation 1	Exponential	NA	1.9 ± 0.2	NA	1.3 ± 0.2	0.80 ± 0.03
Simulation 2	Exponential	NA	1.8 ± 0.2	NA	1.2 ± 0.2	0.82 ± 0.01
Simulation 3	Exponential	NA	2.2 ± 0.2	NA	1.4 ± 0.2	0.83 ± 0.05
Simulation 4	Exponential	NA	1.6 ± 0.2	NA	1.1 ± 0.2	0.83 ± 0.02

This table summarizes the exponents for all of the recordings and simulations. τ, α and β_*S*_ and β_*T*_ are calculated using the scaling procedure described in Section 2. In some cases, the power-law behavior was too limited in range (less than one decade) to reliably estimate the exponents, explaining the absence of a fitted curve. γ is calculated using least-squares fitting on the log of avalanche sizes and durations, where we use 95% confidence bounds to estimate the error.

Our analysis of the experimental data shows in particular that the statistical behavior of the neuronal avalanches in the down state can be statistically indistinguishable from the up state, especially if one focuses on τ, see Figure 3 and Table 2. Note that the different ranges in duration and sizes (see also Supplementary Table S1) are due to the shorter relative duration of the down states with respect to their corresponding ISI, as a comparison with the case of experimental recordings with continuously low steady-state activity (which can be interpreted as a system without an up state and instead being exclusively in a down state) (Yaghoubi et al., 2018) confirms.

**Figure 4:**
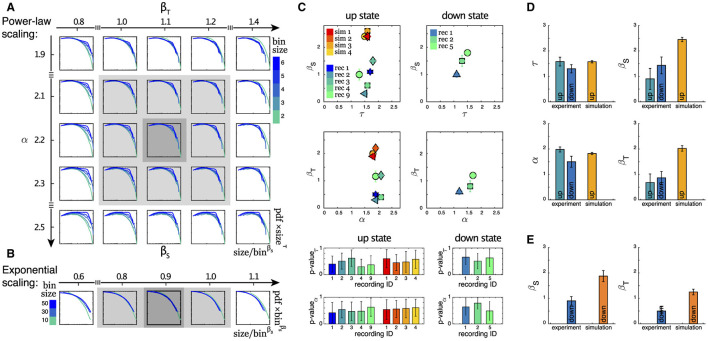
**(A)** The power-law scaling collapse procedure for finding the exponents is visualized for an example experimental dataset (recording 3, up state, avalanche duration). For details of this procedure, see Section 2. **(B)** The exponential-like scaling collapse procedure for finding the scaling exponent β for a sample experimental data is visualized (recording 3, down state, avalanche sizes). **(C)** Summary of the exponents for size and duration of all recordings during up and down states are presented here. Each point represents an experimental or a simulated dataset. We have only included recordings that exhibit power-law behavior over extended ranges as indicated in Table 2. *p*-values of the fitted power laws with exponents τ and α, respectively, are shown in the bottom panels (mean + std of the ensemble of subpopulations). For each of the two columns, the same color scheme as the top panel is preserved. **(D)** Average of all power-law exponents for the data sets in **(C)**. **(E)** Average of β_*S*_ and β_*T*_ for all data sets that show exponential-like behavior as the example in **(B)**.

As outlined in the Section 2, in this study we take advantage of a scaling analysis that also yields critical exponents denoted as β_*S*_ and β_*T*_ that can capture finite size behavior. This provides us with an additional tool for characterizing the dynamics of neuronal systems within the framework of neuronal avalanche statistics. As depicted in Figures 4A, B (see also Supplementary Figure S7), we studied the scaling properties of two types of distributions: (i) Power-law scaling which gives us β_*S*_ (β_*T*_) and τ (α), and (ii) Exponential-like scaling that gives us only β_*S*_ (β_*T*_), for avalanche sizes (durations). The estimate of the exponents becomes more reliable when the scaling collapse is obtained for a wider range of varying bin sizes. The summary of all estimated exponents for (i), along with the corresponding *p*-values for the reported critical exponents τ and α, (see Section 2 for details) for both up and down states, are plotted in Figure 4C. While the variation in τ and α is rather small, especially in the up state, this is not the case for β_*S*_ and, to a lesser degree, for β_*T*_. This higher variability is also visible in Figure 4D, which displays the corresponding mean values and uncertainties for all exponents for both the up and down states. Figure 4E displays the mean values of β_*S*_ and β_*T*_ for distributions exhibiting exponential-like scaling (ii), which exclusively occurs in the down state, see Table 2.

### 3.2 Model simulations

The similarity between the up state and the down state in terms of the neuronal avalanches for our experimental recordings suggests that either (a) there are different mechanisms of information processing in this neuronal system with different associated time scales, or (b) the concept of neuronal avalanches is not specific enough to establish insight into the differences between up and down states. To investigate this further, we employed several computational models that try to mimic the behavior and dynamics of neuronal cultures as described in Section 2.

In the literature, various computational models exist to describe up and down state dynamics. From mostly theoretical (Millman et al., 2010), to those mimicking the conditions of anesthetize animals (Holcman and Tsodyks, 2006), sleep (Bazhenov et al., 2002), or acute slices (Jercog et al., 2017; Camassa et al., 2022) among others. For the case of cultures from dissociated neurons, the up and down dynamics are better described with their own model. This is because in dissociated cultures, a down-to-up state transition can only occur after enough time has passed for the system to recover from a previous up state (Opitz et al., 2002). Once the system has recovered, spontaneous activations can be quickly amplified in a feedback loop due to the presence of recurrent connections, as described by Orlandi et al. (2013). On the other hand, transitions from up to down state are mediated primarily by the depletion of neurotransmitters caused by the high-frequency firings during the up state (Staley et al., 1998; Opitz et al., 2002). The models used here are able to reproduce most of these macroscopic observables as Figure 2 shows. However, when applying the same methodology to characterize the avalanche statistics as in the experimental data, there are important differences. In the simulations, the avalanches observed during the up states followed power-law distributions with slopes of τ≃−1.5 and α≃−1.8 for sizes and durations, respectively, see Figure 3B left and Table 2. These avalanche statistics also satisfied the scaling relation (Equation 12) within the statistical uncertainties (Supplementary Figure S6). During the down state, however, the avalanche statistics were always far from a power-law distribution, suggesting a sub-critical or exponential-like behavior instead (see Figure 3B right). As a result, the down state in the simulated data is absent from Figure 4D.

Although the scaling exponents τ and α were largely consistent during the up states with those observed experimentally (see Table 2), the scaling exponents β_*s*_ and β_*T*_ differed substantially from the experimental ones as follows from Figures 4D, E. The measured exponents across the different simulation conditions were robust, with little variability. The presence or absence of inhibitory connections, as well as heterogeneous cell populations (see Section 2), produced no significant differences in the avalanche statistics. Changes in connectivity strength, synaptic depression parameters (depression strength and recovery time constant), and the presence of other synaptic currents (NMDA), produced no changes in the reported exponents either (not shown). Similarly, although the metric properties of the network structure have a large impact on the presence of the up down transition (Orlandi and Casademunt, 2021), changes in connectivity, i.e., between metric-embedded to a random graph, also resulted in no changes in any of the exponents.

## 4 Discussion

In this work, we present for the first time the simultaneous characterization of neuronal avalanches in dissociated neuronal cultures during both up and down states. In several cultures, the avalanche statistics during both up and down states show critical behavior, as indicated by avalanche sizes and durations. In addition, the associated critical exponents satisfy the corresponding scaling relation (Equation 12), supporting the presence of critical behavior. These exponents are largely within the range of those of a critical mean-field branching process, in line with those previously reported across many preparations (Beggs and Plenz, 2003; Friedman et al., 2012; Yaghoubi et al., 2018). The exponents associated with finite-size scaling show some differences between up and down states. Yet, the variations are large and the statistics is rather limited such that it is difficult to make any definite statements. More importantly, we find that the scaling relation β_*T*_ = γβ_*S*_ (which follows directly from Equation 11) holds within the statistical uncertainties in almost all cases (Supplementary Figure S6), providing further evidence of criticality. The mechanisms by which these cultures can self-organize to maintain critical avalanche statistics across very different activity regimes are still unknown. If differences in the exponents were indeed present, this would further indicate that the two activity regimes are associated with distinct universality classes.

Of particular note is the fact that the temporal bins or time scales necessary to establish critical behavior based on neuronal avalanches are significantly different between up and down states. The time scales are closely related to the average interspike interval per neuron, being significantly larger for the down state. As the example of Figure 3A shows, the relative difference between the temporal bin sizes for neuronal avalanches showing critical behavior between up and down states largely corresponds to the relative difference in the average interspike interval per neuron (see Table 1). While in non-biological systems adjusting the temporal bin sizes over large ranges to evaluate avalanching or information spreading is quite common (Notarmuzi et al., 2022), the range considered for neuronal avalanches is rather limited, typically covering 2–20 ms (see, e.g., Pasquale et al., 2008; Friedman et al., 2012), which is comparable with the time scale of glutamatergic synaptic transmission (Ivenshitz and Segal, 2010). This is consistent with our time bins in the up state (see, for example, Figure 3A, Supplementary Figures S3, S5). Yet, for the down state the time bins necessary to recover neuronal avalanches exhibiting critical behavior are of the order of hundreds of ms (see, for example, Figure 3A, Supplementary Figure S4), suggesting communication mechanisms other than fast glutamatergic synaptic transmission, like those mediated by AMPA receptors, could be at play.

This hypothesis is consistent with our model findings. There has been extensive theoretical and modeling work to try and describe the up and down states as an emergent property of neuronal systems. However, only a few models can simultaneously reproduce the experimentally observed avalanche statistics and switch between up and down states. These include integrate-and-fire networks with structured connectivity through learning (Scarpetta and Candia, 2014); networks with scale-free connectivity coupled to a global field for the up and down switching (Lombardi et al., 2014); self-organized criticality around a saddle-node bifurcation (Millman et al., 2010); and a mesoscopic model on the verge of a synchronization transition (Santo et al., 2018). However, only Millman et al. (2010) treated the avalanche statistics of up and down states separately. They introduced a self-organized critical model with noise-driven, spontaneous transitions between up and down states. The transitions occur around a bifurcation such that the down state is subcritical and the up state is critical. Such a framework, however, is not applicable to our experimental system since its predictions are inconsistent with several experimental observations. Namely, (i) up states possessing characteristic durations, with a well-defined mean and variance (see, for example, Figure 2); (ii) the down states presenting a well-defined duration (usually called the interburst interval, IBI) that is correlated with the slow time-scale of synaptic depression (Cohen and Segal, 2009; Opitz et al., 2002); and (iii) the activity during the down states being possibly critical, as reported here and in Yaghoubi et al. (2018). Despite trying to take all of this into account, our computational model was still unable to reproduce some of the (new) experimental observations. Although the model was able to successfully capture many of the features of spontaneous activity in neuronal cultures (Orlandi et al., 2013), e.g., up and down transition statistics, distribution of states durations and their temporal correlations, up state exponents, etc., it could neither reproduce critical behavior observed during down states nor the values of the finite-size scaling exponents associated with the up states. For the model to simultaneously reproduce critical avalanche statistics during up and down states, its phase diagram would need to have critical points at two very different levels of average activity. The mechanism by which the model would be able to capture this is still unknown.

Conceptually, the fact that these cultures can dynamically transition between different levels of activity and still remain critical (or critical-like), suggests that we might have to move away from the traditional picture of a critical mean-field branching process, and even one of self-organization around a single critical point (Zapperi et al., 1995). Since the observed avalanche statistics need to be defined using substantially different time bins between the up and down states—which is related to their significantly different interspike intervals per neuron quantifying their activity levels—a model that can dynamically adapt the time-scales of synaptic integration (to maintain a constant effective probability of inducing firings from a neuron to their neighbors) could be a good candidate. Glial cells are known to modulate synaptic plasticity across different time-scales (Sancho et al., 2021) and have recently been shown to be involved in neural communication across long time-scales (Mu et al., 2019), hence being a likely candidate to support multi-scale critical dynamics. Investigating this hypothesis remains an exciting challenge for the future.

## Data Availability

The datasets presented in this study can be found in the following online repository: https://github.com/ComplexNSlab/multistateAvalanches.
