# Supplementary material for: Criticality and universality in neuronal cultures during “up” and “down” states
Source: Front Neural Circuits. 2024 Sep 10;18:1456558. doi: 10.3389/fncir.2024.1456558 (PMC11423291; doi:10.3389/fncir.2024.1456558)
Supplement: Supplementary file 1 [file Data_Sheet_1.PDF]

Supplementary Material

1 SUPPLEMENTARY TABLES AND FIGURES

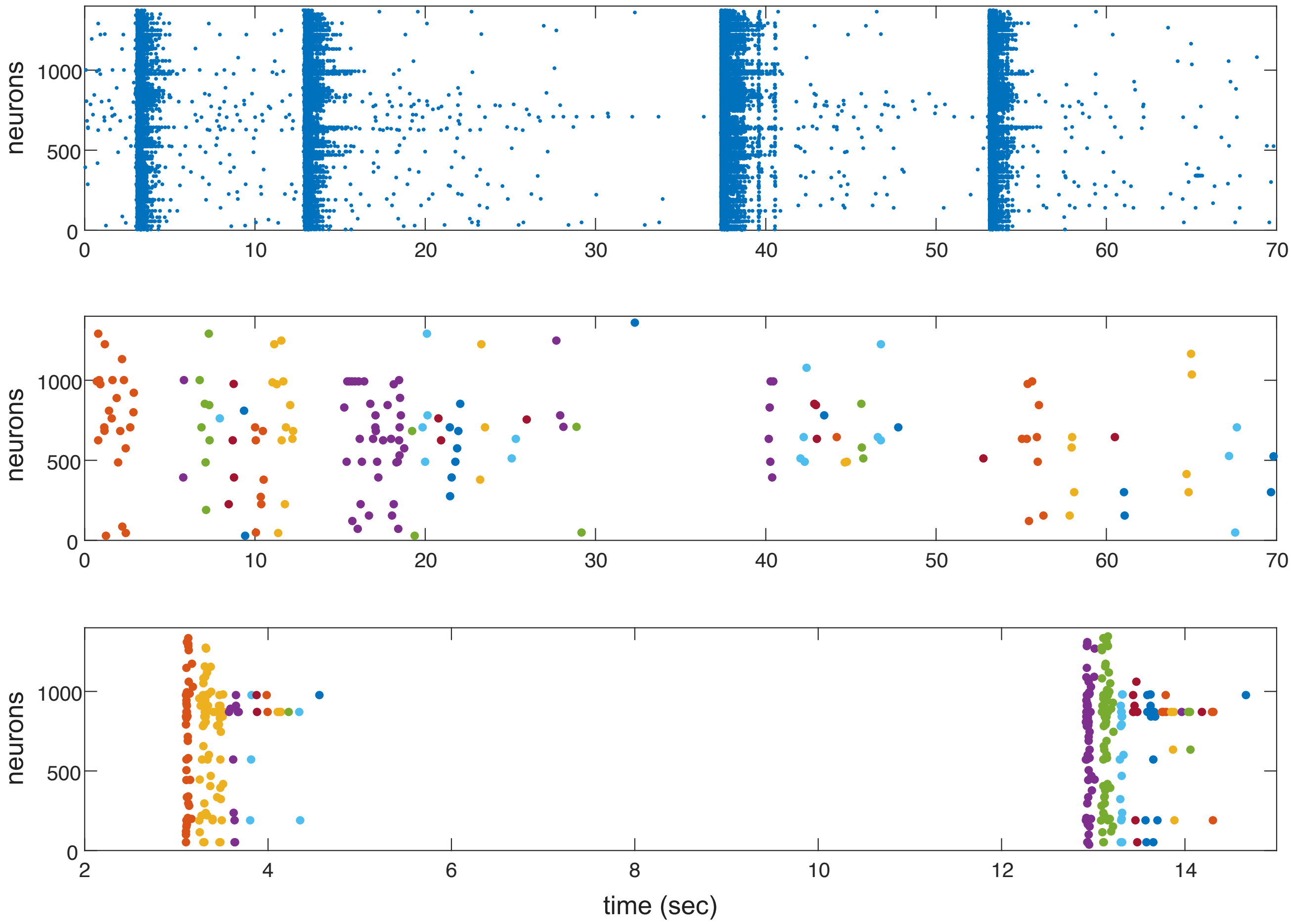

Figure S1. Examples of neuronal avalanches. Top panel: Spike raster plot showing both up and down states. Note that 50% of the neurons are (randomly) selected to define neuronal avalanches (see section 2.7). Middle panel: Neuronal avalanches indicated by different colors during down state only. The chosen temporal bin size is 40 frames (each frame corresponds to 5ms). Bottom panel: Neuronal avalanches indicated by different colors during up state only for the first two up states in the top panel — note the change in the time axis compared to the other two panels. The chosen temporal bin size is 4 frames (each frame corresponds to 5ms).

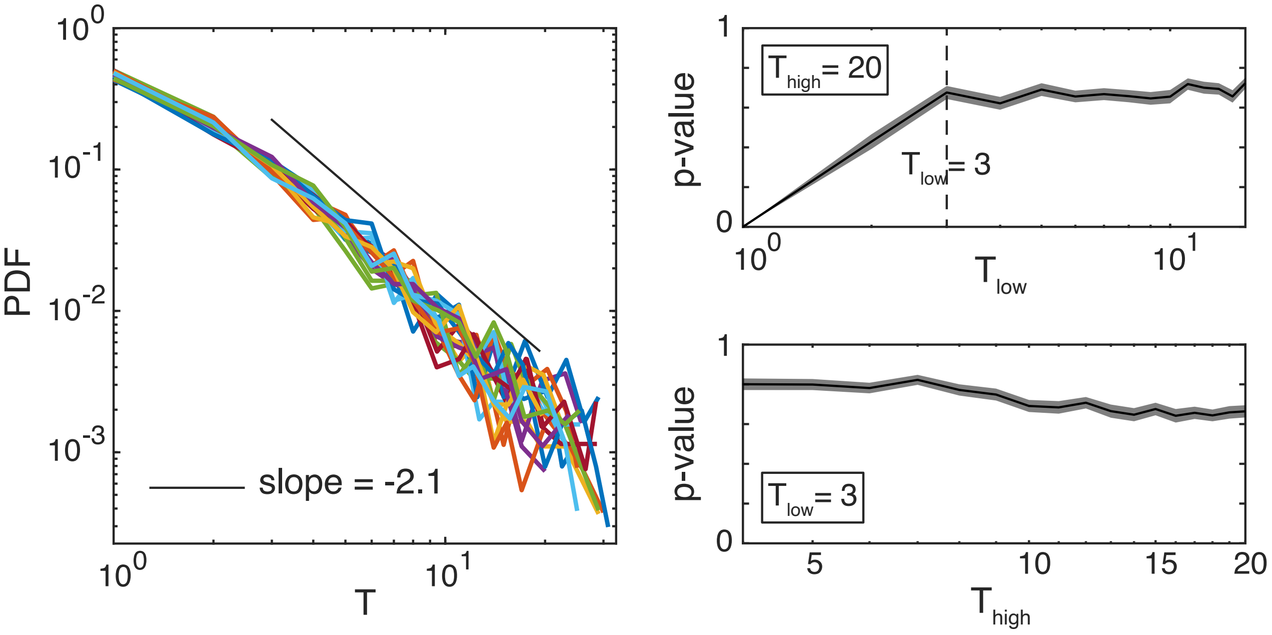

Figure S2. Procedure for estimating the p-value and determining the range of the power-law fit. The procedure for estimating the p-value and determining the range of the power-law fit is as follows, using an example experimental recording: In the left panel, we display the probability distribution of the avalanche durations for 20 randomly selected subpopulations of neurons. The black line superimposed on this panel represents the curve fitted using the scaling procedure explained in the main text. To identify the optimal range with the highest p-value, we follow these steps: As depicted in the top-right panel, we start by manually selecting an upper bound (T_{high}) and then assess the p-value while varying the lower bound (T_{low}). The p-value is calculated using KS statistics as in Equations (7) and (8) from the main manuscript. Notably, we observe that $T_{low} = 3$ marks the point at which the p-value stabilizes at a reasonably high value. Subsequently, as illustrated in the bottom-right panel, we fix the lower bound at the previously determined value and vary the upper bound to identify the largest range that maintains a high p-value. In this particular case, the upper bound can extend to the maximum allowable value, approximately 20. Therefore, the resulting range is [3, 20], with a corresponding p-value of 0.66. In both right panels, the black line represents the mean, and the shaded area indicates the standard error of the mean (SEM) across 100 iterations.

Table S1. Power-law fits: Ranges and p-values.

experiment - up state

	avalanche size range	p-value	avalanche duration range	p-value
rec 1	[4, 40]	0.40±0.30	[1, 4]	0.42±0.38
rec 2	[1, 100]	0.51±0.30	[1, 4]	0.54±0.31
rec 3	[3, 30]	0.62±0.31	[3, 20]	0.66±0.35
rec 4	[3, 50]	0.30±0.28	[1, 5]	0.48±0.36
rec 9	[2, 40]	0.37±0.34	[3, 20]	0.63±0.34

experiment - down state

	avalanche size range	p-value	avalanche duration range	p-value
rec 1	[10, 100]	0.66±0.28	[3, 30]	0.64±0.31
rec 2	[5, 60]	0.66±0.29	[3, 11]	0.78±0.26
rec 5	[3, 30]	0.44±0.32	[2, 12]	0.49±0.35

simulation - up state

	avalanche size range	p-value	avalanche duration range	p-value
sim 1	[2, 200]	0.45±0.30	[2, 50]	0.56±0.32
sim 2	[2, 200]	0.60±0.34	[1, 40]	0.54±0.37
sim 3	[2, 200]	0.58±0.34	[1, 100]	0.61±0.32
sim 4	[2, 200]	0.48±0.39	[1, 50]	0.58±0.35

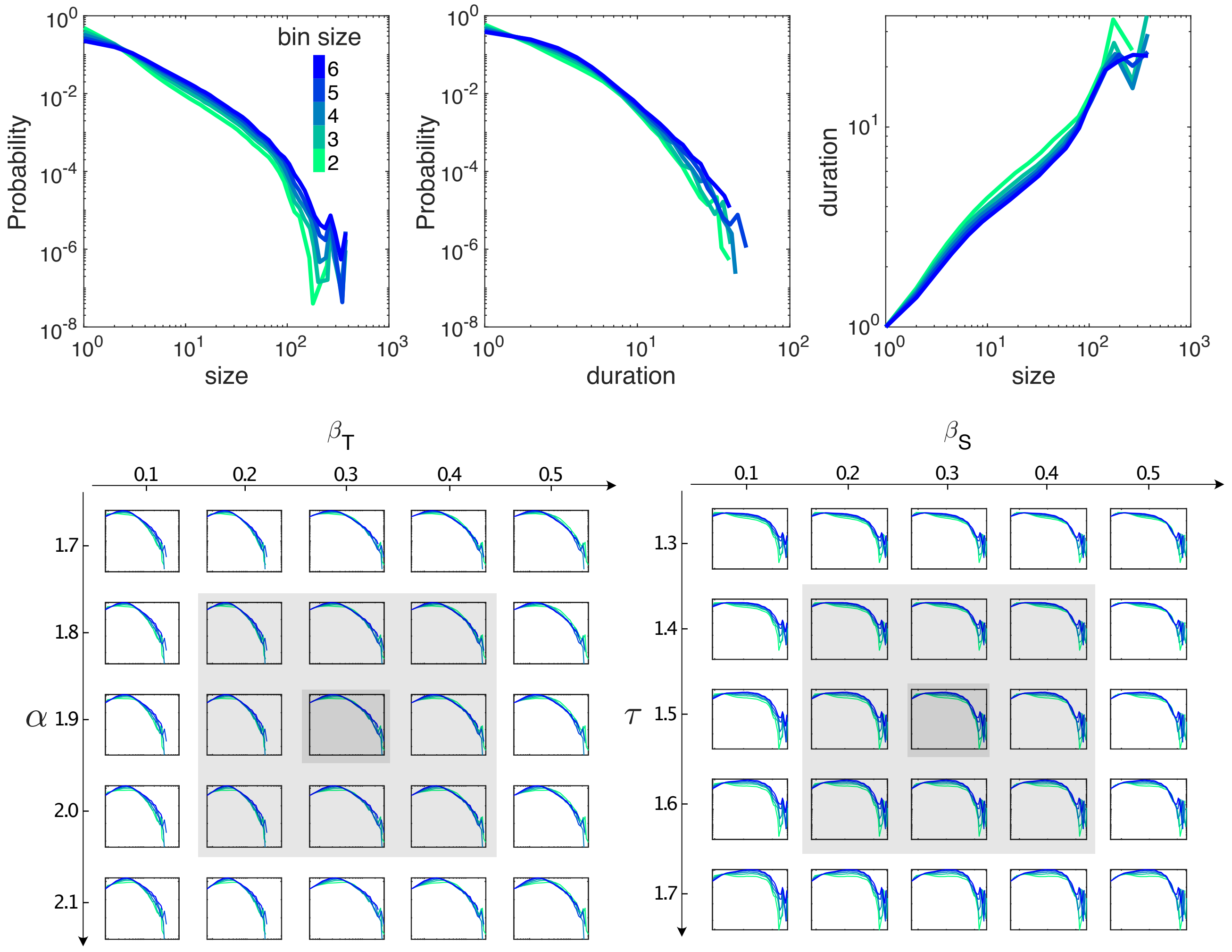

Figure S3. Distributions of neuronal avalanche sizes and durations. Up state of experimental recording 4 for different temporal bin sizes, where the bin sizes correspond to the temporal resolution of the recordings (5 ms).

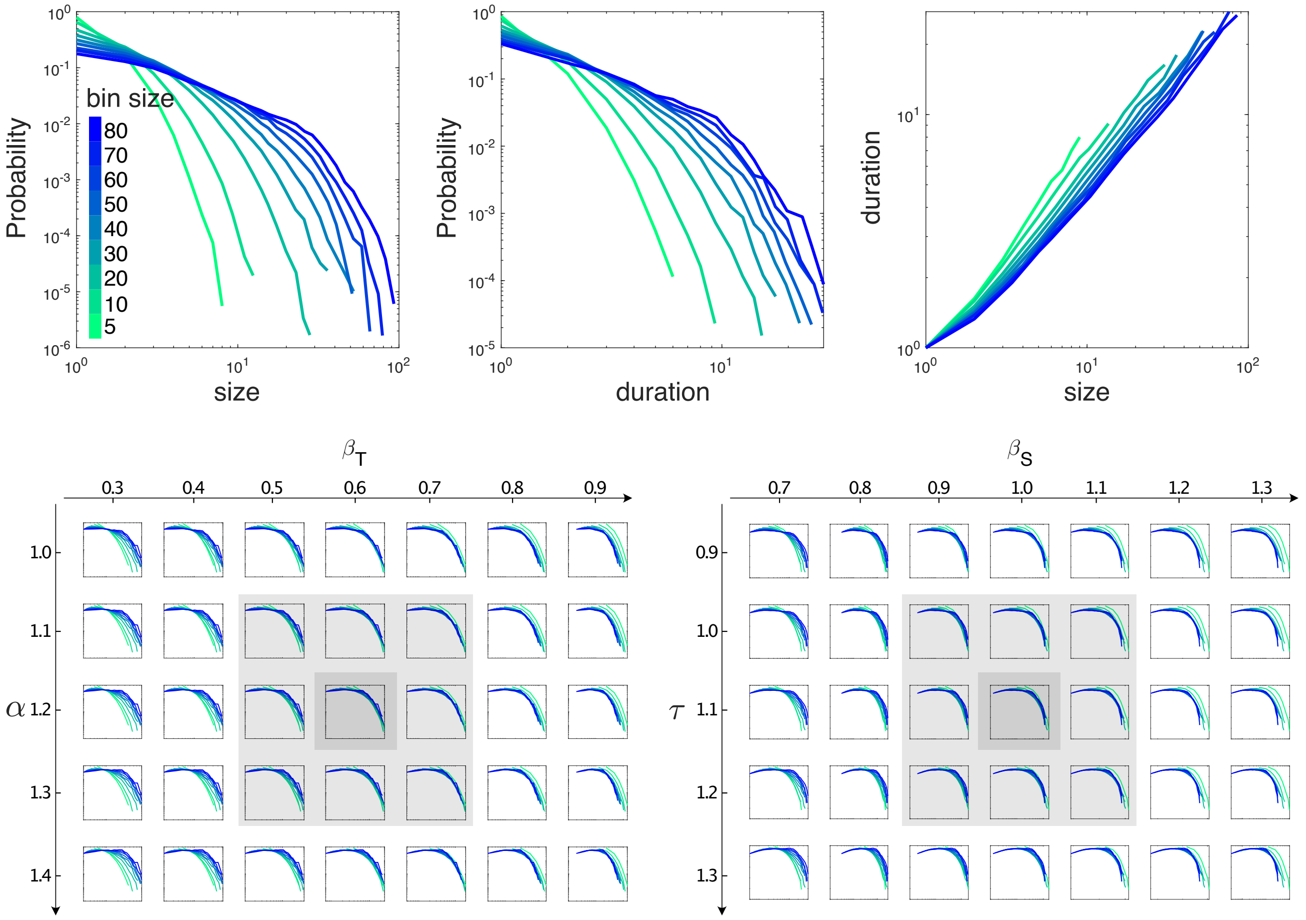

Figure S4. Distributions of neuronal avalanche sizes and durations. Down state of experimental recording 5 (with PTX) for different temporal bin sizes, where the bin sizes correspond to the temporal resolution of the recordings (5 ms).

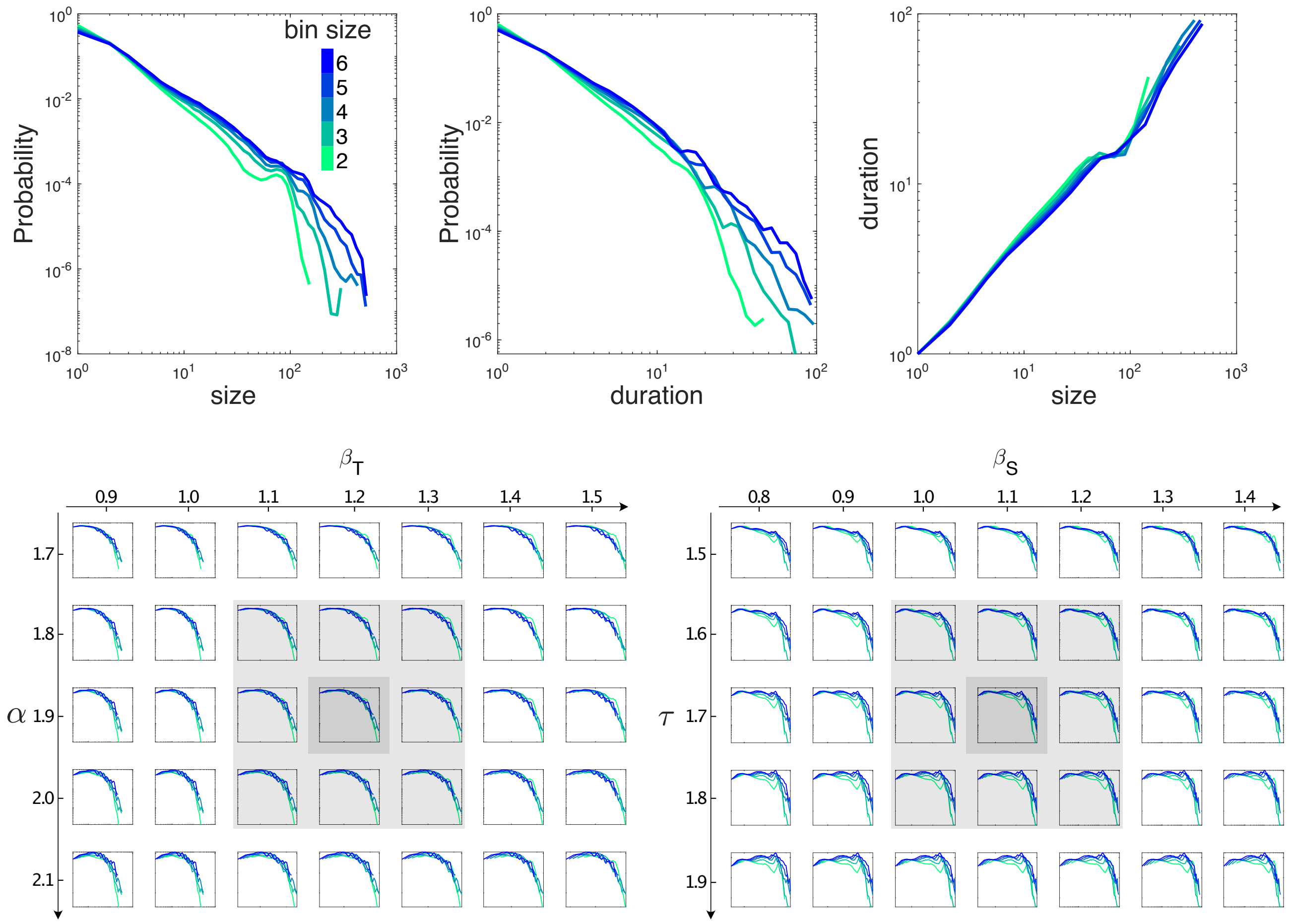

Figure S5. Distributions of neuronal avalanche sizes and durations. Up state of experimental recording 9 (with PTX) for different temporal bin sizes, where the bin sizes correspond to the temporal resolution of the recordings (5 ms).

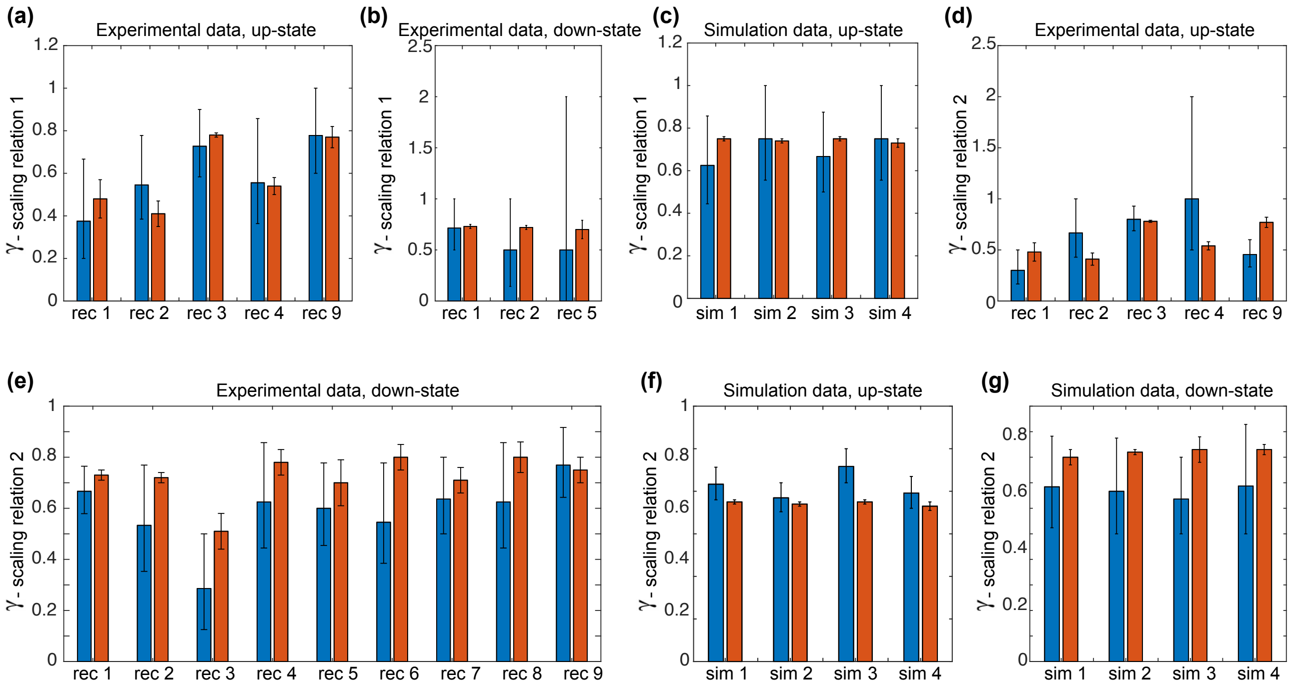

Figure S6. Scaling relations. (a), (b), and (c) test the first scaling relation: $\gamma = (\tau - 1)/(\alpha - 1)$, for experimental down and up states, and simulation up state data, respectively. Blue bars correspond to the predicted values of γ using the scaling relation (the error bars arise from the uncertaintites in α and τ), and orange bars correspond to the directly measured values of γ, see Table 2 in the main paper. Both values agree within the statistical uncertainties in all cases. Similarly, panels (d), (e), (f), and (g) test the second scaling relation: $\gamma = \beta_T/\beta_S$, for experimental up and down states, and simulation up and down states, respectively.

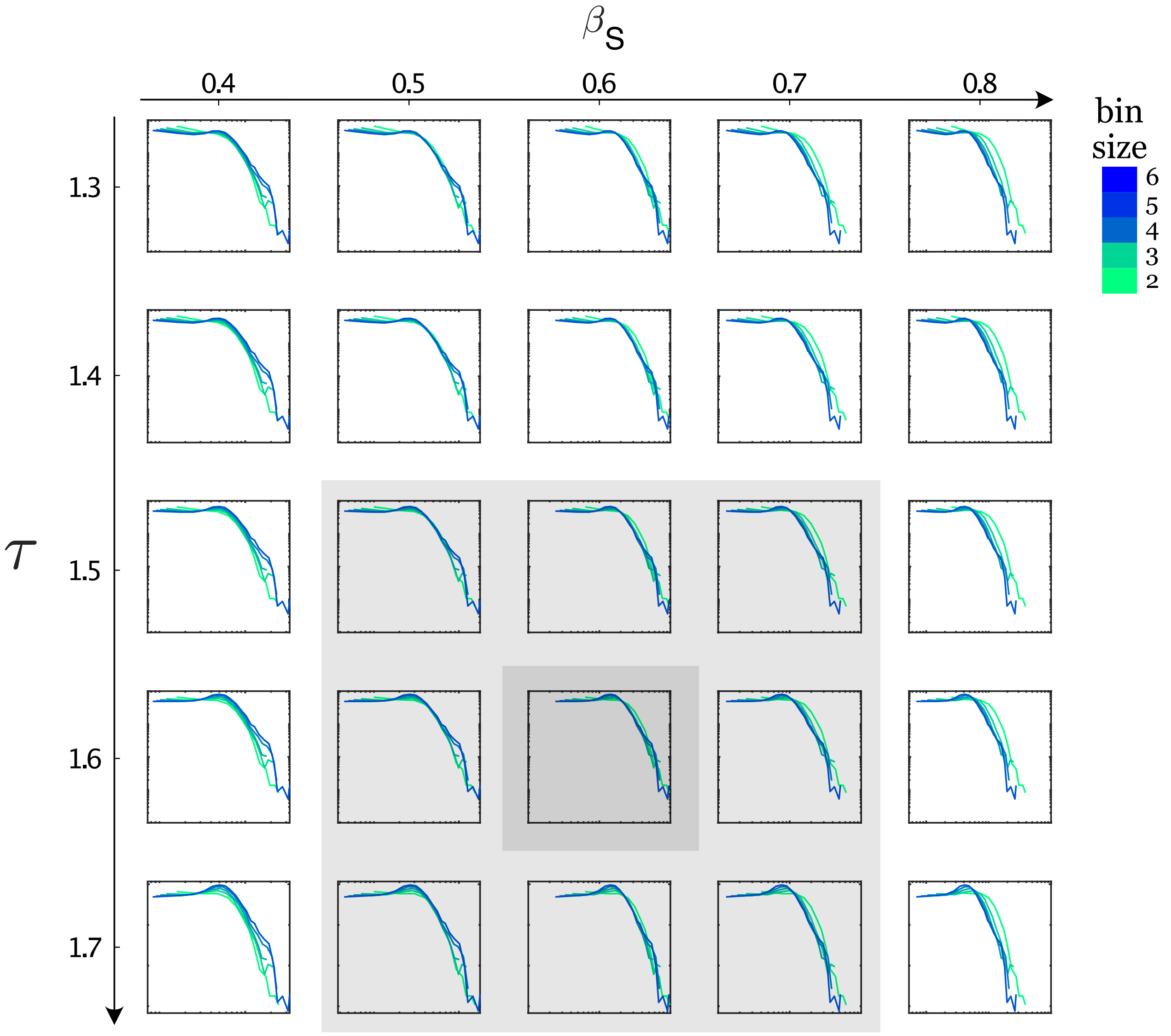

Figure S7. Power-law scaling collapse. Procedure for finding the exponents is visualized for an example experimental dataset (recording 3, up state, sizes) similar to Fig. 4(a). For details of this procedure, see *Materials and Methods*.
